# Use of intravenous lidocaine for dose reduction of propofol in paediatric colonoscopy patients: a randomised placebo-controlled study

**DOI:** 10.1186/s12871-021-01525-0

**Published:** 2021-12-01

**Authors:** Wenshui Yao, Longxin Zhang, Guolin Lu, Jing Wang, Li Zhang, Yuping Wang, Peihan Xiao, Xiaofen Chen, Chanjuan Chen, Min Zhou

**Affiliations:** Department of Anesthesiology, Fujian Maternity and Child Health Hospital, Affiliated Hospital of Fujian Medical University, 18 Daoshan Road, Fuzhou, 350001 Fujian Province China

**Keywords:** Lidocaine, Propofol, Colonoscopy, Children, Adverse events

## Abstract

**Background:**

Propofol, a widely used sedative in endoscopic procedures, sometimes causes cardiopulmonary complications. Intravenous lidocaine can diminish visceral pain and decrease the dose of propofol. The purpose of this study was to assess the efficacy and safety of intravenous lidocaine in reducing propofol dosage during paediatric colonoscopy.

**Methods:**

Forty children who underwent colonoscopy were divided into two groups. Lidocaine hydrochloride (1.5 mg/kg induction and 2 mg/kg/h maintenance) was given intravenously to the lidocaine group, and the same amount of saline was given to the control group after they received lidocaine induction. Propofol initial plasma concentration of 5 μg/mL was targeted, and the procedure was performed after the bispectral index value reached 55. The primary outcome was propofol requirement.

**Results:**

The propofol requirement in the lidocaine group was decreased by 35.5% (128.6 ± 30.4 mg vs. 199.4 ± 57.6 mg; *p* < 0.001; 95%CI: − 100.60, − 41.02). The incidence of involuntary body movements was significantly lower in the lidocaine group (*p* = 0.028; OR = 0.17; 95%CI: 0.03, 0.92). The awakening time (*p* < 0.001; 95%CI: − 7.67, − 5.13) and recovery times (*p* < 0.001; 95%CI: − 7.45, − 4.35) were significantly lower in the lidocaine group. Pain was significantly less at 30 min and 60 min after the procedure in the lidocaine group (0 [0–4] vs. 3 [0–5], *p* < 0. 001; 0 [0–2] vs. 1 [0–3], *p* = 0.001). There was no difference in the incidence of bradycardia, hypotension, or hypoxia between the two groups.

**Conclusions:**

For colonoscopy procedures in paediatric patients, intravenous lidocaine reduces the amount of propofol needed, provides better sedation and postprocedural pain management, as well as a reduction in recovery time.

**Trial registration:**

The trial was registered on November 6, 2020 at China Clinical Trials Registration Center (www.chictr.org.cn) ref.: ChiCTR 2,000,039,706.

## Introduction

Endoscopy is a common evaluation method for a variety of intestinal disorders in children. Although paediatric patients can undergo colonoscopy without sedation [1], it is rarely done so as they often do not cooperate at the same level as adults under “conscious sedation”. Additional personnel may be necessary for procedures involving younger children in order to restrain them, as well as deeply sedate them [2]. Such a level of sedation might increase the risk of airway obstruction and unexpected complications, eventually leading to the need for general anaesthesia [3, 4].

One of the most common sedatives used in outpatient examinations is propofol, due to its rapid effect and short half-life. However, it can cause adverse cardiopulmonary reactions [5]. The combination of midazolam and opioids can be used instead of propofol to reduce these effects, but they can lead to respiratory depression [6, 7].

The bispectral index (BIS) is an electroencephalogram-based monitor, used to provide cerebral pharmacodynamic feedback. Fluctuations in BIS values have been associated with propofol sedation in children over 2 years of age [8, 9]. Using manually infused propofol with clinical symptom guidance in these patients has been shown to have a higher risk of incorrect dosing, in terms of both excess and deficiency, than BIS guidance [10]. A previous preliminary study has shown that a BIS of 50–60 meets the necessary value for paediatric upper gastrointestinal endoscopy [11].

Under the guidance of BIS, target-controlled infusion (TCI) and manual infusion are viable methods to achieve propofol intravenous anaesthesia [10]. Compared to traditional intermittent single doses, propofol TCI has been shown to have a lower cardiovascular and respiratory inhibitory effect [12]. In paediatric propofol intravenous anaesthesia, although TCI does not minimise the dose or decrease recovery time, it may be an easier and safer titration method [13].

Lidocaine is an intravenous adjuvant anaesthetic used for sedation, analgesia, and the suppression of hyperalgesia [14]. A recent study has shown that intravenous injection of lidocaine can be used with propofol to reduce its dosage in adult patients undergoing colonoscopy [15]. A continuous paediatric intravenous infusion of lidocaine-assisted anaesthesia (1–2 mg/kg induction, 1–2 mg/kg/h maintenance) has not resulted in any clinical side effects [16, 17].

There have been no studies conducted on the use of adjuvant lidocaine on paediatric patients undergoing endoscopy. This study aimed to observe the effects of adjuvant lidocaine on propofol, the adverse effects, as well as the postoperative recovery of paediatric patients undergoing painless colonoscopy.

## Materials and methods

### Patients

After obtaining written informed parental consent, children aged 5–12 years who were scheduled for colonoscopy were eligible for inclusion. Only patients classified as physical status I or II by the American Society of Anaesthesiologists (ASA) were included in this study. Patients with obesity, renal failure, hepatic failure, epilepsy, severe arrhythmia, intraoperative therapeutic manipulation (polypectomy), simultaneous gastrocamera examination and allergy to propofol or lidocaine were excluded. This study was conducted at the Fujian Provincial Maternity and Children’s Hospital in Fujian, China, from December 2020 to May 2021.

### Study design

All children were sedated by the same anaesthesiologist and underwent colonoscopy by the same endoscopist. Neither of these physicians were aware of the patients’ distribution group. The children were randomly assigned to one of two groups using the digital table method. The children in the lidocaine group received 1.5 mg/kg of lidocaine intravenously before induction of anaesthesia and were maintained at 2 mg/kg/h until the end of the procedure. The control group received saline in the same volume as the lidocaine group before induction of anesthesia and during procedure. The children in both groups were given 0.01 mg/kg atropine intravenously before induction of anesthesia.

The propofol TCI (Paedfusor model) initial plasma concentration of 5 μg/mL was targeted and the procedure was performed after the BIS value reached 55. The blood pressure was noninvasively monitored every 5 min. Electrocardiography, heart rate (HR), and pulse oximetry (SpO_2_) were continuously monitored.

The research drug was prepared by a nurse anaesthetist who was not involved in patient care or the collection of research variables. Propofol TCI infusion rate was adjusted with a range of 0.5 μg/mL in response to BIS variability exceeding the set range of 50–60, distressed facial expression or hemodynamic changes (HR increase of ≥20 beats/min or systolic blood pressure (SBP) increase of ≥20%). The infusion of propofol was stopped after the operation. During the sedation period, all patients maintained spontaneous breathing and oxygen inhalation of 4 L/min via the nasopharynx. In the event of SpO_2_ level < 90% lasting for more than 10 s, the airway was opened with the jaw-thrust manoeuvre to support the mandible and relieve hypoxia. Atropine 0.01 mg/kg intravenously was planned in the event of bradycardia.

### Measurements

The primary endpoint was propofol requirement, and the secondary endpoints were changes in blood pressure and HR during anaesthesia. Hypotension was defined as a decrease in SBP by more than > 30%. Bradycardia was defined as HR < 60 beats/min in children > 6 years old and HR < 80 beats/min in children ≤6 years old. Hypoxia (SpO_2_ desaturation < 90% lasting for more than 10 s), awakening time (amount of time needed to awaken by command and shoulder tapping after the procedure), recovery time (time from the end of the procedure to a Steward Recovery score of ≥4), and facial visual analogue scale (F-VAS) pain scores were assessed and recorded at 30 min and 60 min after the procedure. The physicians who participated in the evaluation were not aware of the group the child belonged to.

### Statistical analysis

A local pilot study determined that the dosage of propofol in paediatric colonoscopy was 238 ± 70 mg per 30 min. For a 30% difference in propofol requirement between the two groups at alpha = 0.05, and power = 0.90, a sample size of 18 children per group was estimated, resulting in the inclusion of a total of 40 patients. The data were analysed using the SPSS software (version 23; SPSS Inc., Chicago, IL, USA). The parametric data of this study were represented as mean ± SD, while non-parametric data were presented as median (interquartile range). Categorical variables were expressed as percentages. The two-sample t-test was used for continuous parametric data and the chi-squared or Fisher exact test for categorical variables. The Mann–Whitney U test was used for nonparametric data and the repeated observation data were analysed using repeated-measures analysis of variance (ANOVA). Statistical significance was set at *p* < 0.05.

## Results

### Patients’ characteristics

**Fig. 1:**
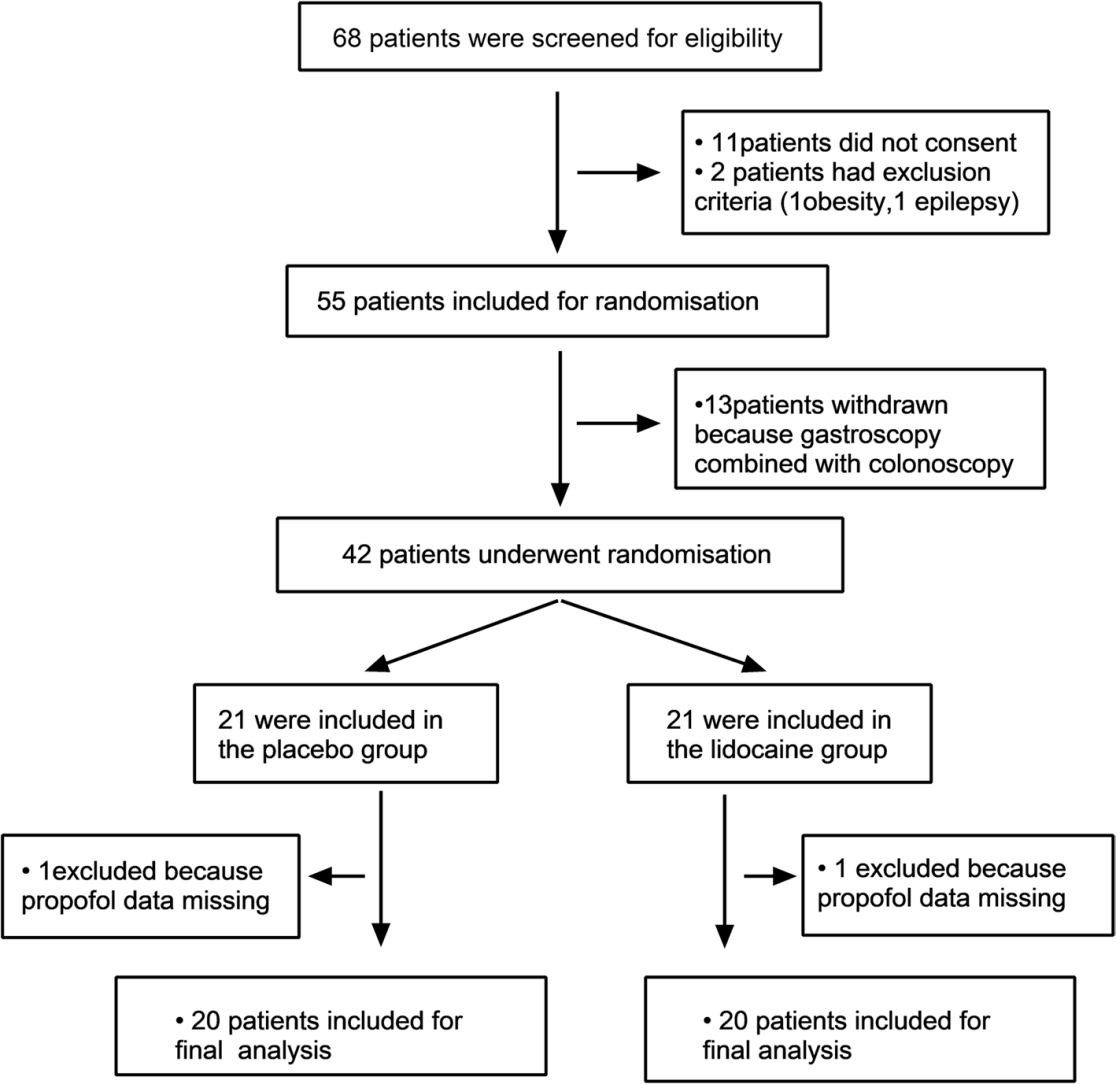
CONSORT trial flow diagram

A total of 40 children participated in this study and were divided into two groups of 20 individuals (Fig. 1). There were no statistically significant differences in age, sex, height, weight, and ASA status between the two groups (Table 1).

**Table 1:** Demographics

Variable	Control group	Lidocaine group	*P* value
Patients, n	20	20	
Age, year	7.1 ± 1.9	7.5 ± 2.1	0.481
Sex, n			0.311
Male	15	12	
Female	5	8	
Weight, kg	23.2 ± 5.2	24.9 ± 5.7	0.329
Height, cm	122.0 ± 11.1	124.7 ± 12.4	0.465
ASA			> 0.999
I	19	18	
II	1	2	
Body mass index	15.4 ± 1.2	15.8 ± 1.1	0.272
Diagnosis, n			0.311
Inflammation/Erosion	8	5	
Other	12	15	

Data are presented as mean ± SD or numbers

### Propofol consumption, sedation time, and adverse events

Intravenous lidocaine reduced the dose of propofol needed by 35.5% (*p* < 0.001; 95%CI: −100.60, − 41.02) in paediatric patients undergoing colonoscopy. This decrease was mainly due to the reduction of the maintenance dose. There was no significant difference in the induction dose between the two groups. Awakening (*p* < 0.001; 95%CI: − 7.67, − 5.13) and recovery times (*p* < 0.001; 95%CI: − 7.45, − 4.35) in the lidocaine group were significantly shorter than in the control group. The number of involuntary body movements in the lidocaine group was significantly less than in the control group (*p* = 0.028; OR = 0.17; 95%CI: 0.03, 0.92). None of the patients in either group required tracheal intubation or supraglottic airway ventilation. Hypotension, bradycardia, and hypoxia occurred in both groups, but there was no significant difference between them. The children with bradycardia were given intravenous atropine 0.01 mg/kg. No serious cardiopulmonary complications were observed in either group (Table 2).

**Table 2:** Propofol usage, time parameters, and incidence of adverse events

Variable	Control group	Lidocaine group	OR	*P* value (95% CI)
Propofol induction dose, mg	63.2 ± 10.1	57.4 ± 11.6		0.112(−13.05, 1.42)
Propofol maintenance dose, mg	136.2 ± 49.6	71.2 ± 26.7		<0.001(−90.78, −39.22)
Total propofol dose, mg	199.4 ± 57.6	128.6 ± 30.4		<0.001(−100.60, −41.02)
Operation time, min	25.9 ± 7.0	24.0 ± 5.8		0.357(−6.03, 2.23)
Awakening time, min	11.2 ± 2.4	4.8 ± 1.4		<0.001(−7.67, −5.13)
Recovery time, min	13.7 ± 2.7	7.8 ± 2.1		<0.001(−7.45, −4.35)
Involuntary movement n (%)	8 (40)	2 (10)	0.17	0.028 (0.03, 0.92)
Hypotension n (%)	3 (5)	2 (10)	0.63	>0.999 (0.09, 4.24)
Bradycardia n (%)	1 (5)	3 (15)	3.35	0.605 (0.32, 35.36)
Hypoxia n (%)	2 (10)	1 (5)	0.47	>0.999 (0.04, 5.69)

Data are presented as mean ± SD or number(%); *OR* Odds ratio, *CI* Confidence interval; *P* < 0.05 denotes statistical significance

### Change in vital parameters

**Fig. 2:**
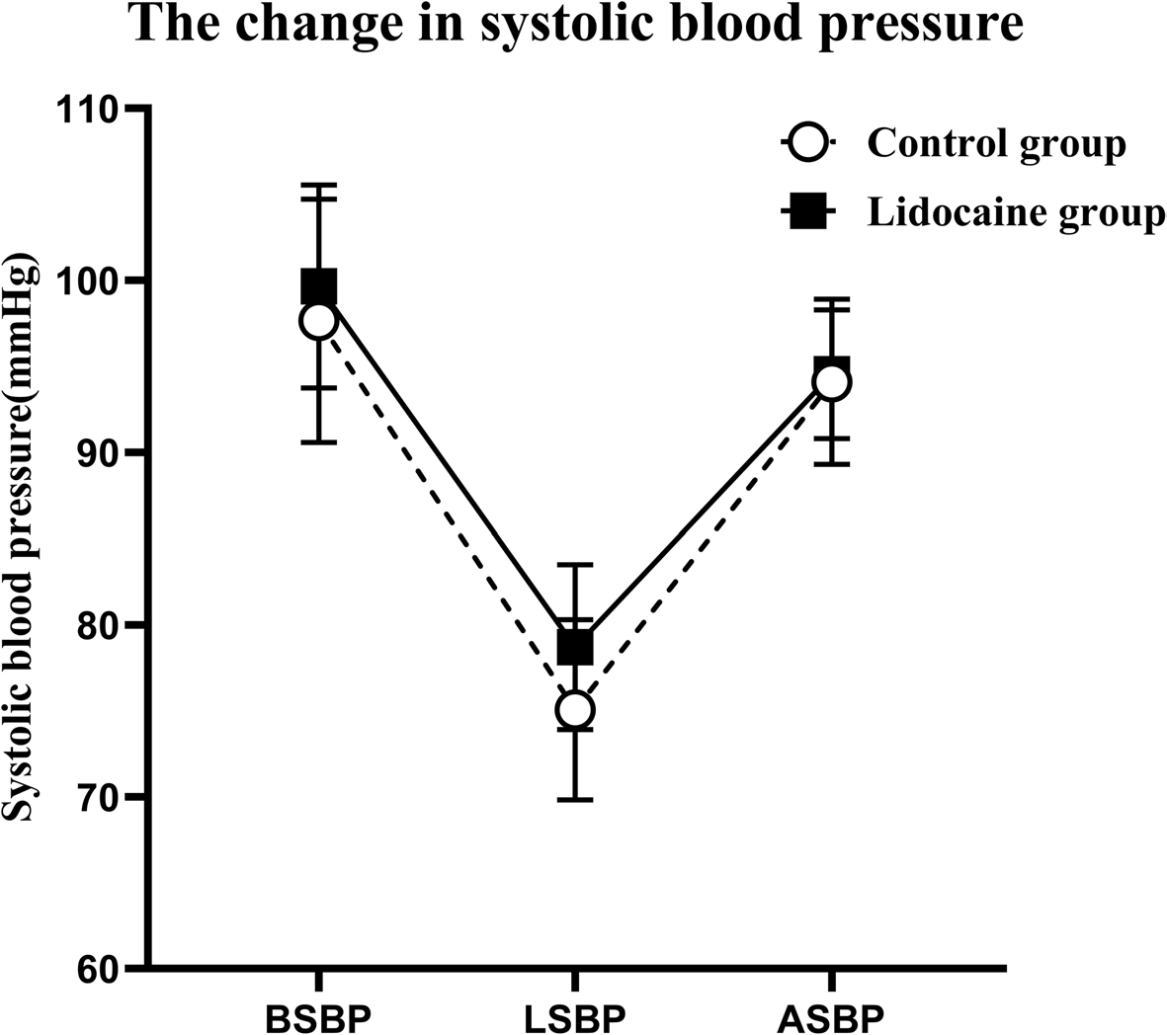
The changes of mean SBP in two groups. The basal SBP (BSBP), the lowest SBP in the procedure (LSBP), and the SBP after the procedure (ASBP). Data are shown as the mean ± SD. No significant difference was detected in blood pressure between the lidocaine group and control group (analysis of variance: *P* = 0.1)

**Fig. 3:**
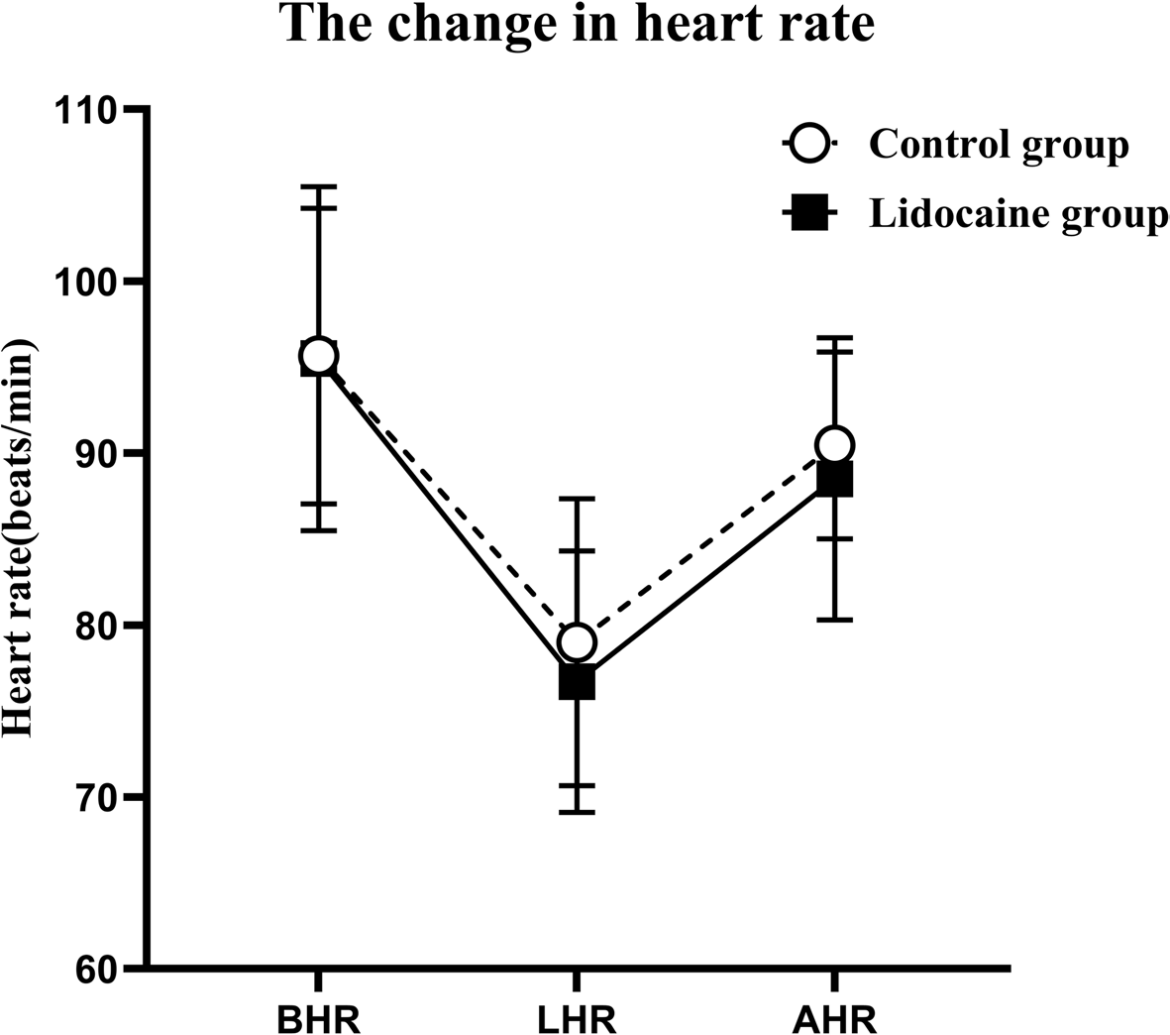
The changes of mean HR in two groups. The basal HR (BHR), the lowest HR in the procedure (LHR), and the HR after the procedure (AHR). Data are shown as the mean ± SD. No significant difference was detected in heart rate between the lidocaine group and control group (analysis of variance: *P* = 0.354)

There were no significant statistical differences in the changes of blood pressure between the two groups [ANOVA: drug effect (df = 1, F = 2.984): *p* = 0.1]; time effect (df = 2, F = 306.886): *p* < 0.001; interaction (df = 2, F = 1.495): *p* = 0.237](Fig. 2). Similarly, there were no significant statistical differences in the changes of heart rate between the two groups [ANOVA: drug effect (df = 1, F = 0.903): *p* = 0.354); time effect (df = 2, F = 74.957): *p* < 0.001; interaction (df = 2, F = 13.308): *p* = 0.6](Fig. 3).

### Post-procedure pain evaluation

**Fig. 4:**
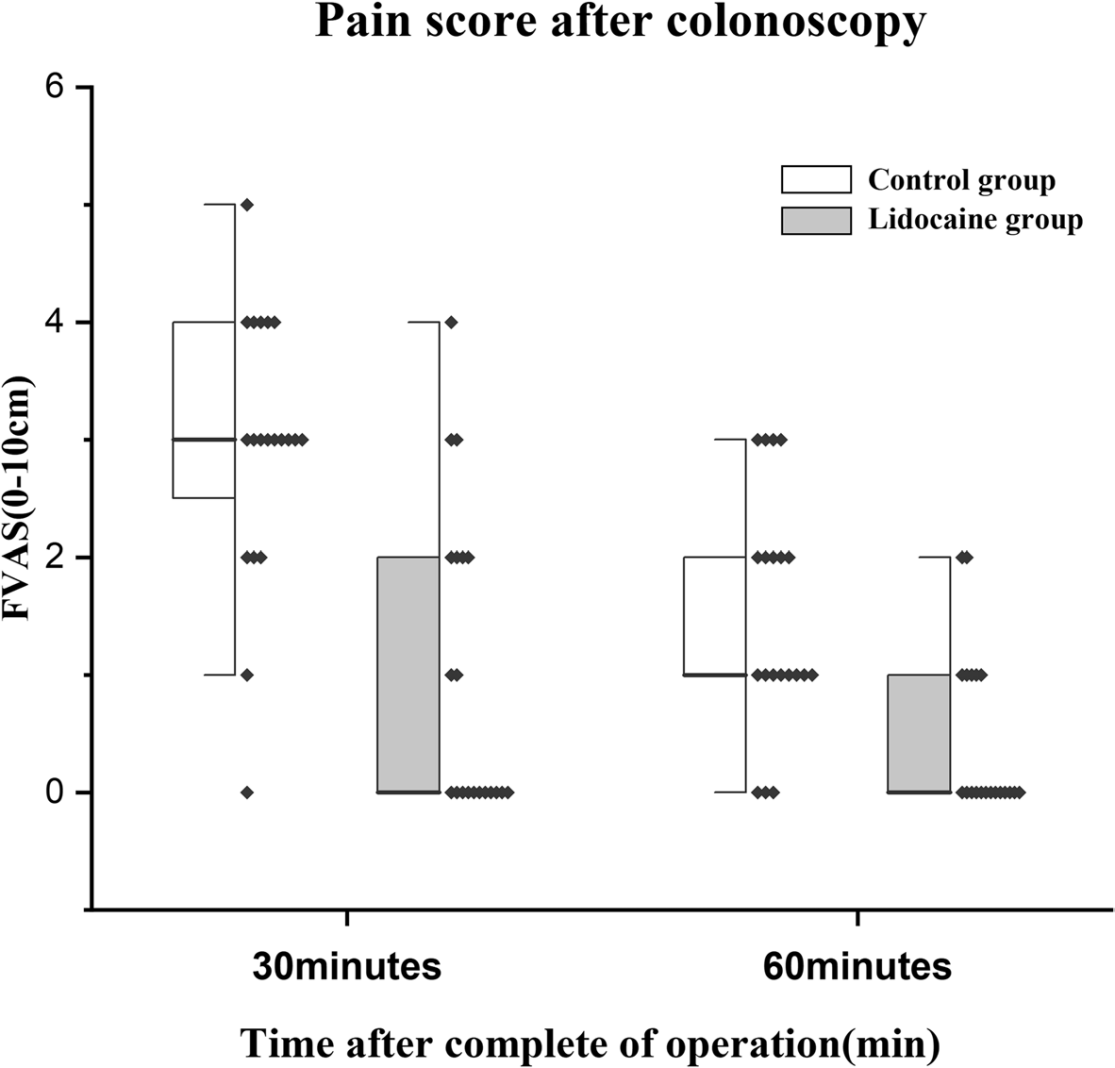
Pain scores after the procedure. Data are shown as the median (range)

The F-VAS pain score 30 min after the procedure in the lidocaine group was significantly lower than in the control group (0 [0–4] vs. 3 [0–5], *p* < 0.001). This was observed after 60 min as well (0 [0–2] vs. 1 [0–3], *p* = 0.001) (Fig. 4).

## Discussion

This study showed that intravenous lidocaine can be safely used in paediatric patients undergoing colonoscopy, reducing the required propofol by 35.5%. Awakening and recovery times were significantly reduced in the lidocaine group, as well as the number of involuntary body movements. Patients in the lidocaine group had significantly lower pain scores after colonoscopy than those in the control group.

The discomfort associated with colonoscopy is primarily due to visceral injury, followed by colon dilation and traction. Intravenous administration of lidocaine has been shown to be effective in reducing visceral pain [18, 19]. It should be noted that in our study, the propofol dose reduction did not sacrifice the working conditions of the endoscopist. On the contrary, the use of lidocaine reduced the number of involuntary movements, providing a better procedural environment.

Another intravenous anaesthetic, ketamine, has been shown to have a good analgesic effect, fast awakening time, in addition to reducing the amount of propofol needed as well as cardiopulmonary complications [20–22]. However, it has been shown to have psychological side effects such as nightmares, hallucinations, and fears, which increase the difficulty of postoperative management, affect the mental health of children and increase the medical costs [23].

The effects of propofol on hemodynamics and respiratory depression were dose-dependent, and the reduction of propofol dose could lower the occurrence of cardiopulmonary adverse events [24]. In addition, relevant studies showed that reduce the dosage of propofol could shorten the recovery time, which was consistent with the results we obtained [25].

Hypoxia and respiratory depression are occasional cardiopulmonary complications associated with endoscopy. Although there was a marked propofol synergistic effect in the lidocaine group, there was no significant difference in the decreased oxygen saturation between the two groups. In our study, the incidence of hypoxemia was approximately 10%, but in previous studies of adults it was higher, reaching 25% [15]. One of the reasons could be because we used a nasopharyngeal tube, which is superior to the nasal cannula [26]. Moreover, we used TCI combined with BIS to stabilise the blood concentration of propofol, reducing excessive anaesthesia.

In our study, we found that as the endoscope was passing through the colonic splenic flexure, the vital signs fluctuated easily, SBP and HR increased to varying degrees, and the children were more likely to have involuntary body movements. This may be due to the anatomical structure that makes it difficult for the endoscope to pass through. Therefore, the appropriate addition of propofol prior to reaching the colonic splenic flexure may reduce the occurrence of this phenomenon.

It should be pointed out that the rate of bradycardia was not significantly different between both groups due to the use of atropine before induction to prevent the sharp fluctuation of HR caused by propofol.

Our study found that the pain score after colonoscopy in the lidocaine group was significantly lower than in the control group. Postoperative pain can make children nervous, scared, and restless, which can have a long-term psychologic impact [27]. The use of lidocaine can reduce postoperative pain and may be more beneficial for the postoperative management of these patients.

One of the limitations of this study is the lack of transcutaneous carbon dioxide monitoring. Although there was no difference in the oxygen saturation between the two groups, timely monitoring of carbon dioxide accumulation may help in detecting early respiratory inhibition.

In conclusion, intravenous lidocaine significantly reduced the amount of propofol needed in paediatric patients undergoing colonoscopy. The incidence of involuntary movements, the amount of postprocedural pain, and the recovery time were also reduced. Larger trials are required to confirm these results.

## Data Availability

The datasets used and/or analysed during the current study available from the corresponding author on reasonable request.

## Abbreviations

BIS: Bispectral index
TCI: Target-controlled infusion
ASA: American Society of Anaesthesiologists
HR: Heart rate
SpO2: Pulse oximetry
SBP: Systolic blood pressure
F-VAS: Facial visual analogue scale
ANOVA: Repeated-measures analysis of variance
BSBP: Basal SBP
LSBP: Lowest SBP in the procedure
ASBP: SBP after the procedure
BHR: Basal HR
LHR: Lowest HR in the procedure
AHR: HR after the procedure
OR: Odds ratio
CI: Confidence interval
